# Validation of Antimycobacterial Plants Used by Traditional Healers in Three Districts of the Limpopo Province (South Africa)

**DOI:** 10.1155/2013/586247

**Published:** 2013-07-14

**Authors:** Peter Masoko, Kulani Mashudu Nxumalo

**Affiliations:** University of Limpopo, Department of Biochemistry, Microbiology and Biotechnology, Private Bag X1106, Sovenga 0727, South Africa

## Abstract

The aim of the study was to scientifically evaluate the antimycobacterial activity of selected indigenous medicinal plants from the Limpopo Province used for the treatment of humans with symptoms of *Mycobacterium tuberculosis*. The leaves of five plant species (*Apodytes dimidiata, Artemisia, Combretum hereroense, Lippia javanica, and Zanthoxylum capense*) were collected from the Lowveld National Botanical Garden in Nelspruit, South Africa. The dried leaves were powdered and extracted using hexane, dichloromethane, acetone, and methanol. Antimycobacterial activity was evaluated using microdilution assay and bioautography and **ρ**-iodonitrotetrazolium violet (INT) as indicator. Antioxidant activities were determined by 2,2-diphenyl-1-picrylhydrazyl (DPPH). Phytochemical content of extracts was further evaluated. The acetone extracts of *L. javanica* displayed antioxidant activity on BEA chromatogram. T Acetone extracts of *A. afra* had MIC value of 0.39 mg/mL against *Mycobacterium smegmatis* ATCC 1441. Acetone extracts of *C. hereroense* and *L. javanica* had MIC value of 0.47 mg/mL. Four bands that inhibited the growth of *M. smegmatis* were observed at *R*_*f*_ values of 0.12, 0.63, and 0.87 on BEA and 0.73 on EMW. The plant species *A. dimidiata, A. afra, C. hereroense,* and *L. javanica* in this study demonstrated their potential as sources of anti-TB drug leads.

## 1. Introduction

Diseases and other related ailments are inevitable in life and have led man to discover ways by which they could be treated. Plants have always been a successful source of remedy from nature. Such practice is as old as human existence and forms an integral part of traditional medicine. Medicinal plants are simply defined as those plants, that can elicit therapeutic properties and induce health towards man and animals. These could include any part of the plant that is, roots, stems, leaves, bark, seeds, fruits, and flowers which can be composed of the right constituent to restore to health. The products can be ingested in their natural state or be prepared in a ready to use form [1]. Human Immunodeficiency virus/acquired immune deficiency syndrome (HIV/AIDS), malaria, diabetes, sickle-cell anemia, mental disorders, and microbial infections are illnesses whereby medicinal plants have dominantly played a vital role in their treatment. The most important advantages in using medicinal plants are that they are easily available than synthetic alternatives, yielding profound therapeutic benefits, and this is a more inexpensive treatment [2]. 

South Africans have a long history of the use of medicinal plants in treating a variety of illnesses and ailments [3]. Medicinal plants have always played a significant task within the traditional health care system of South Africa. Moeng [4] estimated that in 1994 between 12 and 15 million or 60% of the people of South Africa used medicinal plant remedies from as many as 700 indigenous species. The average South African consumer of traditional medicine uses 750 g of plant material a year. With the widespread use of medicinal plants by indigenous people, the search for biologically active agents based on traditional use is still relevant as these plants have the potential to provide pharmacologically active compounds [3]. The lack of adequate information with regard to dosage taken by adults and given to children poses a serious challenge as medicinal plants contain other compounds which are biologically active and cause adverse effects due to their toxicity [5].

Street et al. [6] further report that the following are contributing factors towards variation in biological activity: plant age, seasonal variation, and geographical deviation in harvest sites. Raw plant material undertakes very slight processing before being administered to the patient. Bioactive compounds are not separated from the crude plant material when administering the traditional medicine instead they use entire plant or parts of the plant. Dried plant material is the most common form of traditional medicine with the advantage of increased shelf-life.

Tuberculosis (TB) is an infection primarily affecting the lungs. It is caused by *Mycobacterium tuberculosis* which is acknowledged as a pathogenic bacteria affecting a third of the world's population. One serious challenge in the prevention of TB is that it can be transferred through the air when an affected person can expel the disease by means of coughing. Mativandlela et al. [7] reported that between 2000 and 2020 almost 20 million individuals will fall victims of TB and 35 million will unfortunately die. Tuberculosis largely affects developing countries especially those in Asia and Africa with the highest occurrence in Africa. Challenges accounting for such occurrence are the lack of health facilities necessary to combat the threat of TB or if available they are far away from the individuals affected such as those living in rural settlements [8].

The misuse of antibiotics and not taking an entire course of treatment have led to the recent development of multidrug-resistant TB (MDR-TB) and extensively drug-resistant TB (XDR-TB) which now acts as a serious challenge to the health care system [9]. Fatal cases of TB in the year 2006 were 1.7 million of which 14% were coinfected with HIV. MDR-TB is resistant to rifampicin and isoniazid while demanding chemotherapy that is expensive using drugs said to be toxic. XDR-TB is resistant to rifampicin, isoniazid, and other drugs such as capreomycin, kanamycin or amikamycin and fluoroquinolone [8].

Several researchers for example, Lall and Meyer [10] and Mativandlela et al. [7], have successfully studied some medicinal plants that can be possibly used in the treatment of TB using different techniques. However, the Bapedi treatments were not included. The selected plants were screened because they were used to treat TB-related symptoms. Other activities of the selected plants are indicated in Table 1. The aim of the study was to scientifically evaluate the antimycobacterial activity of selected indigenous medicinal plants from the Limpopo Province which may be used for the treatment of humans infected with *Mycobacterium tuberculosis.*

## 2. Materials and Methods

### 2.1. Plant Collection and Storage

The leaves of *Apodytes dimidiata* E.Mey. ex Arn, *Artemisia afra *Jacq. ex Willd*, Combretum hereroense *Schinz*, Lippia javanica *(Burm.f.) Spreng, and* Zanthoxylum capense* (Thunb.) Harv were collected at the Lowveld National Botanical Garden. Voucher specimens in the garden herbarium and tree labels verified the identity of the plants. Plants were confirmed by Mr. Willem Froneman (Control Horticulturist). He also provided plants accession details (*A. dimidiata* (LNBG 1969/46), *A. afra *(LNBG 2010/27)*, C. hereroense *(LNBG 1977/71)*, L. javanica *(LNBG 1969/460), and *Z. capense *(LNBG 1969/100)). The collection was based on their ethnopharmacological information provided by traditional healers in the Sekhukhune, Waterberg and Capricorn districts of the Limpopo Province. The plants were air dried at the Microbiology Department, University of Limpopo, and the dried leaves were milled using a blender (Waring Laboratory Blender LB20ES) to fine powder and stored in bottles in the dark room until required for extraction to prevent oxidation. Ground fine plant material is the most efficient substance to study plants as fewer challenges are encountered than when using fresh plant material [11]. 

### 2.2. Extraction Procedure

Powder of *A. dimidiata*, *A. afra, C. hereroense, L. javanica and Z. capense* leaves was extracted by weighing 1.0 g of finely ground plant material and extracting it with 10 mL of n-hexane, dichloromethane (DCM), acetone, and methanol in different polyester centrifuge tubes, respectively. Tubes were vigorously shaken for 10 minutes in series 25 shaking incubator machine (New Brunswick Scientific Co., Inc.) at a high speed (100 rpm), and the extracts were filtered into preweighed labelled bottles. The process was repeated three times to exhaustively extract the compounds, and the extracts were combined. The solvent was removed under a stream of cold air at room temperature.

### 2.3. Phytochemical Analysis

The plant extracts were re-dissolved in acetone to give a final concentration of 10 mg/mL. For each plant 10 *μ*L (100 *μ*g) was loaded on aluminium-backed thin layer chromatography (TLC) plate (Sigma), and the plates were developed in saturated chambers with three solvent systems of different polarity, namely, benzene/ethanol/ammonium solution (18 : 2 : 0.2) [BEA] (nonpolar/basic); chloroform/ethyl acetate/formic acid (10 : 8 : 2) [CEF] (intermediate polarity/acidic); ethyl and acetate/methanol/water (10 : 1.35 : 1) [EMW] (polar/neutral) [12]. The plates with the separated compounds were viewed under ultraviolet (UV) light (254 and 365 nm) for compounds which are fluorescing and later sprayed with vanillin sulphuric acid reagent (0.1 g vanillin (Sigma) : 28 methanol : 1 mL sulphuric acid) and heated at 110°C for optimal colour development and visualize colours of the different compounds in each extract. The sprayed plates were scanned with a laser scanner and analyzed. 

### 2.4. Preliminary Biochemical Analysis of Phytochemicals

Acetone plants extracts were tested for the presence of saponin, phlobatannin, tannins, terpenes/terpenoids, steroids, cardiac glycosides, and flavonoids using the standard procedures as described by [2].

### 2.5. Antioxidant Assay

TLC plates were used to separate extracts as above. The plates were dried in the fume-hood. To detect antioxidant activity, chromatograms were sprayed with 0.2% (w/v) 2, 2-diphenyl-2-picrylhydrazyl (DPPH) (Sigma) in methanol as an indicator. The presence of antioxidant compounds was detected by yellow spots against a purple background on TLC plates sprayed with 0.2% DPPH in methanol [13].

### 2.6. Bacterial Species

The test organism* Mycobacterium smegmatis *ATCC 1441 was obtained from the School of Molecular and Cell Biology, University of Witwatersrand. The bacterial species was grown and maintained in Middlebrook 7H9 (Fluka M0178) broth with glycerol (Fluka 49769) or Tween 80 (Fluka 93780) and Middlebrook Oleic Albumin Dextrose Catalase (OADC) growth supplement (Fluka M0553). 

### 2.7. Quantitative Antibacterial Activity

#### 2.7.1. Minimum Inhibitory Concentration (MIC) Determination

The MIC values were determined using the serial microplate method developed by Eloff [11]. Minimum inhibitory concentration is described as the lowest concentration of the compounds inhibiting the growth of microorganisms. Dried extracts were redissolved in acetone to a concentration of 10 mg/mL of crude extracts. The plant extracts were serially diluted 50% with water in 96-well microtitre plates. Bacterial cultures were subcultured and transferred into fresh Middlebrook 7H9 broth and 100 *μ*L of the culture was transferred into each well, and appropriate acetone blanks were included. The microtitre plate was incubated at 37°C for 24 hours. After incubation, 20 *μ*L of *ρ*-iodonitrotetrazolium violet (Sigma) (INT) dissolved in water was added to each of microplate wells as an indicator of growth [11]. The covered microplates were incubated for 30 minutes at 35°C and 100% relative humidity for colour development. All determinations were carried out in triplicate. Microorganism growth led to the emergence of a purple-red colour resulting from the reduction of INT into formazan. Clear wells indicated the presence of compound in the extracts that inhibited the growth of the microorganisms tested. 

#### 2.7.2. Qualitative Antibacterial Activity (Bioautography)

For bioautographic analysis 20 *μ*L of each extract was loaded on the TLC plates. The plates were developed in mobile phases as previously mentioned. The chromatograms were dried at room temperature for about four days to remove the solvents used to develop chromatograms. The chromatograms were sprayed with overnight culture of *M. smegmatis* until completely wet and were incubated at 37°C in a humidified chamber for 24 hours. The plates were sprayed with *ρ*-iodonitrotetrazolium violet (INT) (Sigma) and incubated for a further 24 hours. The presence of clear bands on the plates against a purple background indicated growth inhibition [29].

## 3. Results and Discussion

Medicinal plants serve as a potential avenue for drug discovery to combat diseases and other related ailments. Isolated compounds can serve as precursor constituents for therapeutic drugs. The initial and critical step in isolating the compound of interest is extraction. Four different solvents were used to extract the active compounds from the leaves of *A. dimidiata, A. afra*, *C. hereroense, L. javanica*, and *Z. capense. *The solvents used in the study were hexane, DCM, acetone, and methanol. 

**Figure 1:**
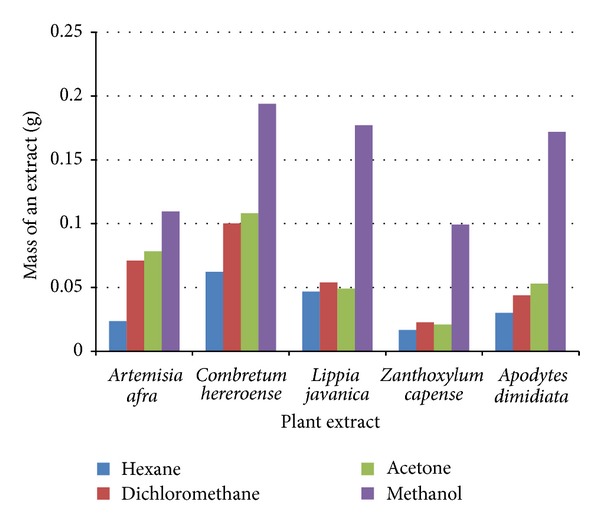
The mass of the plants extracted using different solvents (extraction process).

The four solvents were used to extract a wide range of plant compounds. The most common solvent used by traditional healers is water which is limited by its inability to extract non-polar compounds. Water frequently does not dissolve the intermediate polar to nonpolar components of a dried extract. The success of determining the biologically active compounds largely depends on the type of solvent used in extraction therefore it is important to use solvents that will extract all compounds that is, covering all range of polarity. Methanol was the best extractant resulting in a greater yield of plant material extracted and hexane was the least extractant (Figure 1). Masoko et al. [30] reported methanol extracts had the best extract yield. *Combretum hereroense *had the best extracted material, and *Z. capense* had the least extracted plant material. Following extraction, extracts were redissolved in acetone as reported by Eloff [22] as it has the ability to dissolve many hydrophilic and lipophilic compounds being miscible with water containing the least toxicity effects on both bacterial and fungi species. 

 McGaw et al. [31] report that analysis of the phytochemicals of plant crude extracts can be conducted by thin layer chromatography which offers effectiveness and fast outcome that can be used to obtain the profile of the plant extracts. More bands were observed in BEA, followed by CEF and EMW (results not shown), which indicates that in the selected plant there is an abundance of non-polar compounds. Evaluating the composition of plant extracts is important in trying to elucidate the specific compound that is responsible for therapeutic property hence the need to initially attain the fingerprints of plant extracts.

Antioxidant activity of the extracted compounds was evaluated by using thin layer chromatography (results not shown). The acetone extracts of *L. javanica *showed distinctive band that has antioxidant activity on BEA chromatogram while the rest of the plant extracts failed to display any distinctive separated band that demonstrates any antioxidant activity. This suggests that *L. javanica *contains a compound that has antioxidant activity. Lekganyane et al. [32] reported antioxidant activity in *L. javanica, *and Pretorius [28] also reported that *L. javanica* shows great potential as a medicinal plant with antioxidant activity and may therefore be beneficial in decreasing the negative oxidative effects caused by free radicals. The significance of evaluating antioxidant activity in the plant extracts was to discover any link or relationship between the antioxidant activity and the therapeutic property being investigated. Many plants that possess antioxidant activity have been known to have numerous therapeutic properties. 

 Terpenes/terpenoids and steroids were present in all plants, and saponins were the least present (Table 2). *A. dimidiata* and *C. hereroense* had 5 out of the 7 phytochemicals investigated, and *A. afra* had 4. Flavonoids which have been reported to have numerous beneficial medicinal properties which include antimicrobial and antioxidant activity were present in all the plants. Terpenes/terpenoids are also reported to have medicinal properties such as anti-carcinogenic, antimalaria, antiulcer, antimicrobial and diuretic activities [33]. This suggests the importance of preliminary phytochemical screening when studying medicinal plants and can be meaningful.

The acetone extracts of *L. javanica *displayed antioxidant activity on BEA chromatogram. Acetone extracts of *A. afra* had MIC value of 0.39 mg/mL against *Mycobacterium smegmatis. *Acetone extracts of *C. hereroense* and *L. javanica* had MIC value of 0.47 mg/mL. Acetone was the best extractant, it extracted antibacterial agents which was indicated by the lowest MIC values in all screened plants. The results obtained serve as a scientific validation for the use of the plants in traditional medicine for treatment of TB and other respiratory ailments as well as their efficiency in TB drug discovery. Mmushi et al. [16] also reported the antibacterial activity of *A. dimidiata* against *M. smegmatis*. 

Eloff [22] reports that MIC should not be the only aspect taken into account when assessing the activity of extracts, but the total activity must also be considered. The total activity is calculated as the quantity of material extracted from one gram of dried plant material divided by the minimum inhibitory concentration value [34]. The unit is mL/g and indicate the largest volume to which the biologically active compounds in one gram of plant material can still be diluted and be able to inhibits the growth of the test organism. *Z. capense *extract had the highest average total activity of 94 mL/g followed by *A. dimidiata* with 15 mL/g (Tables 3 and 4). This suggests that the extract prepared from 1 gram of plant material could be diluted to a volume of 94 and 15 mL for *Z. capense* and *A. Dimidiate*, respectively, and still inhibits *M. smegmatis* efficiently. 

**Figure 2:**
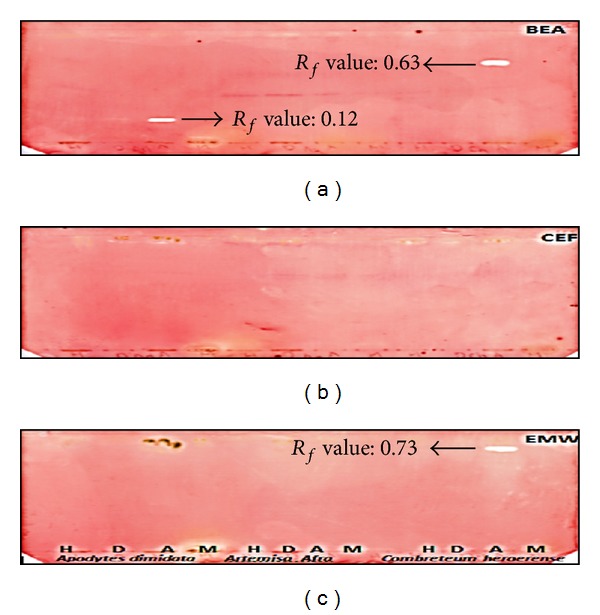
Bioautograms of crude extracts of *A. dimidiata, A. afra,* and *C. hereroense* extracted with hexane (H), dichloromethane (D), acetone (A) and methanol (M) in lanes from left to right for each plant, separated by BEA (top), CEF (middle), and EMW (bottom) and sprayed with *M*. *smegmatis. *White areas indicate where reduction of INT to the coloured formazan did not take place due to the presence of compounds that inhibited the growth of *M*. *smegmatis. *

**Figure 3:**
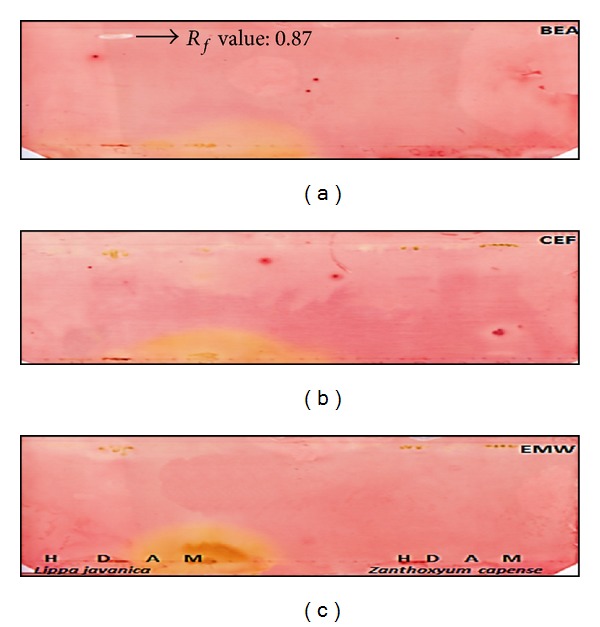
Bioautograms of crude extracts of *L. javanica* and *Z. capense* extracted with hexane (H), dichloromethane (D), acetone (A) and methanol (M) in lanes from left to right for each plant, separated by BEA (top), CEF (middle), and EMW (bottom) and sprayed with *M*. *smegmatis. *White areas indicate where reduction of INT to the coloured formazan did not take place due to the presence of compounds that inhibited the growth of *M*. *smegmatis. *

The plant extracts were analysed by bioautography for qualitative analysis of antibacterial compounds using thin layer chromatography sprayed with *M. smegmatis* (Figures 2 and 3). After a period of 48 hours INT was used as a growth indicator and zones of inhibition were assessed. The acetone extracts of *A. dimidiata *and *C. hereroense* and dichloromethane extract of *L. javanica* demonstrated inhibition of the growth of *M. smegmatis *on the BEA bioautogram. *C. hereroense* displayed one band that inhibited growth in the EMW bioautogram. A total of 4 bands were observed that inhibited growth of *M. smegmatis*. The *R*_*f*_ values were 0.12, 0.63, and 0.87 for *A. dimidiata*, *C. hereroense*,* L. javanica *respectively in the BEA chromatogram. The *R*_*f*_ value for *C. hereroense *in EMW chromatogram was 0.73. The *R*_*f*_ value will assist in isolation and identification of the bioactive compound. 

For the other plant extracts, the acquired ethnopharmacological information provided by traditional healers failed to be scientifically validated by bioautography as the rest of the plants failed to display any activity against *M. smegmatis*. The lack of correlation between the obtained MIC values and bioautography bands can be attributed to the vaporization of volatile active compounds during removal of the TLC eluents or disturbance of synergism between the active constituents caused by TLC separation [35]. The other possible explanations could be that the antibacterial activity which accounts for the anti-TB effect of the plants is mediated by other mechanism rather than direct inhibition on mycobacteria. Some bioactive compounds require activation metabolically *in vivo* by particular enzymes in the cell or their antibacterial activity is largely dependent on the pH of the cells. Therefore, the negative results obtained from this study cannot prevent the potential anti-TB effect of the medicinal plants [36].

## 4. Conclusions

The plant species *A. dimidiata, A. afra, C. hereroense *and *L. javanica *displayed effective antibacterial activity towards* M. smegmatis* demonstrating their potential as sources of anti-TB drug leads. Therefore, the attained association between the supposed main classes of compounds in the extracts and promising activity in the study may lead in the future isolation and antibacterial evaluation of the bioactive compounds. Further phytochemical and pharmacological studies of these plants are essential and significant. 

## Figures and Tables

**Table 1:** Selected plants used by Bapedi traditional healers to treat tuberculosis.

Plants name	Family	Vernacular name	Uses	Reference
*Apodytes dimidiata* E.Mey. ex Arn	Icacinaceae	Sephopha-madi	Molluscicide for snail control in antischistosomiasis programmes in rural communities	[14]
			Antiprotozoal activity, haemolytic activity, and antiangiogenic activity	[15]
			Antimicrobial activity	[16]

*Artemisia afra* Jacq. ex Willd	Asteraceae	Lengana	Hypertension and related conditions	[17]
			Coughs, colds, sore throat, heartburns, haemorrhoids, fevers, malaria, asthma, diabetes mellitus, and revealed hepatoprotective effect	[18]
			Cardioprotective effect	[19]
			Antimicrobial, antioxidant, sedative, antidepressant on the CNS, cardiovascular, and spasmolytic activity	[20]
			Antimicrobial activity	[21]

*Combretum hereroense* Schinz	Combretaceae	Mokabi	Anthelmintic activity	[22]

*Lippia javanica* (Burm.f.) Spreng	Verbenaceae	Musukudu or bokhukhwane	Respiratory ailments specifically coughs, colds, and bronchial problems	[23]
			Anthelmintic	[24]
			Respiratory ailments specifically bronchitis, colds, and coughs	[25]
			Antimicrobial activity	[26]
			Antibacterial and antioxidant activities	[27]
			Antioxidant activity	[28]

*Zanthoxylum capense* (Thunb.) Harv.	Rutaceae	Monokwane	Treats sores by the Zulu people and serves as a good mouthwash in case of a toothache	[23]
			Treatment of TB and other respiratory diseases	[9]

**Table 2:** Phytochemical screening of the plant species selected for the study.

Plant name	Saponins	Phlobatannins	Tannins	Terpenes/terpenoids	Steroids	Cardiac glycosides	Flavonoids
*A. dimidiata *	+	−	+	+	+	−	+
*A. afra *	−	−	+	+	+	+	+
*C. hereroense *	−	−	+	+	+	+	+
*L. javanica *	−	−	+	+	+	+	+
*Z. capense *	+	−	+	+	+	+	+

−: not present, +: present.

**Table 3:** Minimal inhibitory concentration (MIC) values (mg/mL) of selected plant species against *M. smegmatis* after 24 hours of incubation at 37°C.

Extractant	Minimal inhibitory concentration (mg/mL)					
	*A. dimidiata *	*A. afra *	*C. hereroense *	*L. javanica *	*Z. capense *	Average
Hexane	n/a	n/a	1.25	0.62	2.5	**1.46**
DCM	0.94	0.62	0.62	1.25	2.5	**1.19**
Acetone	0.62	0.39	0.47	0.47	0.62	**0.51**
Methanol	1.90.	1.25	1.90	1.25	na	**1.5**
Average	**0.78**	**0.75**	**1.06**	**0.90**	**1.87**	

Rifampicin = 125 *µ*g/mL.

Key: no activity (n/a).

**Table 4:** Total activity values (mL/g) of selected plant species against *M. smegmatis* after 24 hours of incubation at 37°C.

Extractant	Total activity (mL/g)					
	*A. dimidiata *	*A. afra *	*C. hereroense *	*L. javanica *	*Z. capense *	Average
Hexane	n/a	n/a	20	13	150	**61**
DCM	21	9	6	23	110	**34**
Acetone	12	5	4	10	30	**12**
Methanol	11	11	5	7	na	**9**
Average	**15**	**8**	**9**	**13**	**97**	

Key: no activity (n/a).
